# Generative AI in lung adenocarcinoma pathology: methodological and interpretive considerations

**DOI:** 10.1097/JS9.0000000000003440

**Published:** 2025-09-05

**Authors:** Wen He, Lin Zhu

**Affiliations:** a Department of Respiratory and Critical Care Medicine, Suzhou Ninth Hospital Affiliated to Soochow University, Suzhou, China; b Central Laboratory, Suzhou Ninth Hospital Affiliated to Soochow University, Suzhou, China

*Dear Editor,*

We read with great interest the recently published article evaluating the application of generative artificial intelligence (GenAI) models in the histopathological assessment and prognostic prediction of lung adenocarcinoma^[1]^. The authors deserve credit for exploring a novel direction in this rapidly evolving field. As GenAI models become increasingly integrated into biomedical imaging, such work is timely and of potential importance. By systematically comparing state-of-the-art vision-capable GenAI systems and extending their application to prognostic modeling, this study provides an ambitious and thought-provoking contribution. All content complies with TATIN guidelines^[2]^.

That said, several aspects warrant careful interpretation. First, the reliance on manually selected regions of interest (ROIs) may underestimate the inherent complexity and heterogeneity of lung adenocarcinoma. In routine pathology, subtle but clinically meaningful patterns often occur within small foci, which may be overlooked when only representative ROIs are sampled^[3]^. Ironically, such an approach may reintroduce observer bias at a stage where AI is expected to provide objective assistance. Without whole-slide image (WSI) analysis, claims regarding generalizability remain constrained. Future evaluations should prioritize WSI-based automated workflows to reduce sampling bias and improve robustness.

Second, the study identifies Claude-3.5-Sonnet as the “best-performing” model, citing higher accuracy and moderate consistency. However, the reported intraclass correlation coefficient of 0.585 would traditionally not be considered strong, particularly in tasks with direct clinical implications^[4]^. Thus, conclusions about “reliable prognostic ability” may be premature. Prognostic signals derived from GenAI features are intriguing, but they need to be benchmarked against established clinical predictors and validated in broader, more heterogeneous cohorts before translational use can be considered.

Third, the study reflects a broader issue in current discussions of GenAI in medicine: enthusiasm sometimes outpaces practical applicability. GenAI systems are not specifically optimized for pathology, and their apparent versatility may conceal vulnerabilities when faced with rare histologic patterns (e.g., micropapillary structures) or real-world artifacts such as tissue folding, necrosis, or staining variability. The brief mention of limitations in detecting micropapillary structures further underscores this point.

Finally, while the authors highlight the potential of GenAI to reduce pathologists’ workload, the study’s dependence on precise ROI extraction implies the opposite – additional manual preprocessing steps may, in fact, increase workload. A more balanced perspective may view GenAI as an adjunct rather than a replacement, with particular utility in standardization and education, but not yet ready to assume independent diagnostic or prognostic responsibilities.

In summary, this study represents an important early exploration of GenAI in lung adenocarcinoma pathology. Nonetheless, its methodological limitations, moderate reproducibility, and interpretive framing call for tempered expectations. Future work should emphasize WSI-based analysis, multicenter validation, and careful benchmarking against existing deep-learning pathology pipelines. Such steps will be essential to translating the promise of GenAI into reliable clinical practice.

## Data Availability

Not applicable.

## References

[1] Shen J Feng S Zhang P. Evaluating generative AI models for explainable pathological feature extraction in lung adenocarcinoma: grading assessment and prognostic model construction. Int J Surg 2025;111:4252–62. 40434749 10.1097/JS9.0000000000002507

[2] Agha R Mathew G Rashid R. Transparency in the reporting of artificial intelligence-the TITAN guideline. Prem J Sci 2025;10:100082.

[3] Kothari S Phan JH Stokes TH Wang MD. Pathology imaging informatics for quantitative analysis of whole-slide images. J Am Med Inform Assoc: JAMIA 2013;20:1099–108. 23959844 10.1136/amiajnl-2012-001540 PMC3822114

[4] Koo TK Li MY. A guideline of selecting and reporting intraclass correlation coefficients for reliability research. J Chiropr Med 2016;15:155–63. 27330520 10.1016/j.jcm.2016.02.012 PMC4913118

